# Treatment of Alveolar Abscess

**Published:** 1869-08

**Authors:** C. S. Chittenden


### ARTICLE VII.

### *Treatment of Alveolar Abscess.*

By C. S. Chittenden.

In my remarks on the treatment of this disease, I propose to confine myself to those cases which arise from the destruction of the nerve, either from exposure from decay, or from the use of devitalizing preparations. The first thing to be done is to discharge the abscess. If no opening has been formed through the alveolus and gums, I clean out the pulp chamber and open the nerve canal, and then pass a small broach up or down, through the apical foramen, (this can be done much more easily after suppuration has taken place than while a devitalised nerve remains in the canal) and allow the pus to escape through the tooth. When all the pus has escaped, that will pass out of itself, I inject tepid water into the canal so as to clear it as thoroughly as possible from all putrifying matter, and close the cavity of decay with cotton dipped in sandarach, and allow it to remain in that condition for a day or two, when I cleanse it again with tepid water, and inject a weak solution of nitrate of silver into it. I use the nitrate of silver for the purpose of *stimulating* the inner surfaces of the abscess to a healthy action ; I then close the cavity again for another day or two with cotton and sandarach, and almost invariably find that nature has cured the disease when I next examine the case. If not, I use a dressing of creosote or carbolic acid and tincture of Iodine for a few days longer. When there is an opening through the gums I open the nerve canal as before, but I also cut down through the gums and alveolus to the apex of the root of the tooth, and discharge the pus from the abscess through the gums. I then endeavor to pass the solution of nitrate of silver through the tooth and out through the external opening which I have made. After filling the nerve canal with cotton to stop any fetid matter from passing into it, I pass a tent of cotton into the external opening so as to prevent its closing, and stopping the discharge from passing out through it. One, two or more dressings may be required, according to the health and vitality of the patient, but I always feel *certain of success* if I have been able to adapt the treatment perfectly, in the manner indicated.

As soon as the tooth appears to be in a healthy condition, that is, when I find that it is firm in its socket, and no pain is produced on tapping it smartly, I, at once proceed to fill the root or roots, and the cavity of decay with gold. I do not think it is necessary to wait for the external opening to heal, as there is very little fear that any trouble will arise, after the nerve canal has been so perfectly closed that nothing can pass into it through the opening at the end of the root. —*Canada Journal of Dental Science.*

